# Shaping Performance of XP4 versus ProTaper Next in C‐Shaped Canals: A Micro‐CT Study of 3D‐Printed Mandibular Second Molar Replicas

**DOI:** 10.1002/cre2.70434

**Published:** 2026-08-13

**Authors:** Zaidao Xiong, Lu Wang, Wenyuan Zhou, Zhongyang Shan, Chao Liu, Yongchun Gu, Jia Li

**Affiliations:** ^1^ Department of Dentistry and Central Lab, Ninth People's Hospital of Suzhou Soochow University Suzhou China

**Keywords:** C‐shaped mandibular second molars, micro‐computed tomography, nicke–titanium rotary system, root canal preparation, three‐dimensional printing

## Abstract

**Introduction:**

This micro‐computed tomography (micro‐CT) study aimed to evaluate the shaping ability of two nickel–titanium (NiTi) rotary systems—XP4 and ProTaper Next (PTN)—in 3D‐printed replicas of C‐shaped canals.

**Methods:**

Two extracted mandibular second molars with C‐shaped root canals—one continuous (Melton's Category I) and one discontinuous (Melton's Category II/III)—were selected and replicated via 3D printing. Replica accuracy was validated by best‑fit alignment. Sixteen resin replicas per specimen were randomly assigned to PTN or XP4 instrumentation (*n* = 8 per group) following the manufacturers' protocols. Pre‐ and post‐instrumentation micro‐CT scans were performed. Three‐dimensional registration and reconstruction enabled quantitative analysis of (1) volume/surface area changes and (2) unprepared canal surfaces.

**Results:**

The replicas demonstrated high fabrication fidelity. In continuous C‐shaped canals, PTN removed significantly less resin (2.7 mm^3^, 7.4% of initial volume) than XP4 (4.3 mm^3^, 11.6%; *p* < 0.01). Conversely, in discontinuous C‐shaped configurations, PTN exhibited greater removal (13.0 mm^3^, 23.1%) versus XP4 (8.9 mm^3^, 15.8%; *p* < 0.01). Unprepared canal surface in continuous C replicas was higher with PTN (42.0%) than XP4 (25.5%; *p* < 0.01), whereas no significant difference was observed in discontinuous C replicas (PTN: 32.0%, XP4: 31.6%; *p* > 0.05). Procedural complications were observed exclusively in discontinuous C‐shaped canals, with PTN causing two cases of critical wall thinning and XP4 exhibiting two instrument separations.

**Conclusion:**

In continuous C‐shaped canal replicas, XP4 showed superior shaping capability with significantly lower percentages of unprepared surface compared to PTN. In discontinuous C‐shaped configurations, both systems achieved comparable unprepared surface areas, although they differed markedly in resin removal patterns and iatrogenic risks. These results indicate that the relative performance and safety profiles of NiTi systems may vary depending on specific C‐shaped canal anatomy. Clinicians should consider canal configuration, curvature severity, and dentin thickness when selecting instruments for C‐shaped canal instrumentation.

## 1 Introduction

The mandibular second molar classically exhibits two distinct roots with two mesial and one distal canal (Tang et al. 2025). A clinically important anatomical variation is the C‐shaped canal configuration, characterized by buccal or lingual root fusion forming a ribbon‐shaped inter‐radicular connection between the canals with isthmus or web‐like structures (Cooke and Cox 1979; Manning 1990; Melton et al. 1991; Jafarzadeh and Wu 2007; Fan et al. 2004). This morphology likely results from incomplete fusion of Hertwig's epithelial root sheath to the buccal or lingual root surface (Manning 1990). The prevalence of C‐shaped canals demonstrates significant ethnic variation, occurring in 10%–40% of East Asian populations but rarely (< 5%) in Black or Caucasian groups (Burse et al. 2024; Martins et al. 2018; Zheng et al. 2011).

Conventional radiography often fails to detect this configuration due to two‐dimensional (2D) imaging limitations, including structural superimposition, geometric distortion, and inadequate contrast resolution, necessitating high clinical suspicion (Jin et al. 2024). Cone‐beam computed tomography (CBCT) offers superior three‐dimensional (3D) visualization and is the diagnostic gold standard (Jin et al. 2024), yet clinical management remains technically demanding. The complex anatomy of C‐shaped canals poses considerable challenges in endodontic treatment, including difficulties in thorough debridement, effective disinfection, and optimal 3D obturation due to irregular canal fins, intricate anastomoses, and variable isthmus morphology (Cheung and Cheung 2008; Baghbani et al. 2021). Furthermore, the associated radicular groove often corresponds to thin dentin, increasing the risk of strip perforation during instrumentation (Cheung and Cheung 2008).

The development of nickel–titanium (NiTi) rotary systems represents a significant breakthrough in endodontic therapy, particularly for managing anatomically complex cases (Zhou et al. 2013; Liang and Yue 2022; Gavini et al. 2018; Bürklein et al. 2013; Elnaghy and Elsaka 2014; Elnaghy 2014; Arias et al. 2014; Velozo et al. 2020; Gagliardi et al. 2015). These innovative instruments combine superior flexibility with shape memory characteristics, enabling more efficient canal preparation while reducing procedural errors such as transportation and ledging. However, persistent limitations include susceptibility to cyclic fatigue failure in severely curved canals and incomplete debridement of inaccessible areas. Contemporary researches have consequently focused on enhancing the mechanical properties of NiTi alloys and refining instrument kinematics to optimize dentin‐instrument interface dynamics. Such technological advancements have demonstrated particular efficacy in improving debridement outcomes for non‐circular canal systems exhibiting oval or flattened configurations, while simultaneously maintaining the critical balance between procedural efficacy and clinical safety.

The ProTaper Next (PTN) system (Dentsply Maillefer, Ballaigues, Switzerland) utilizes M‐Wire NiTi alloy technology to optimize instrument flexibility and resistance to cyclic fatigue. This system incorporates an asymmetric rectangular cross‐section that generates a distinctive swaggering motion, enhancing its ability to follow natural canal pathways while maintaining centering ability (Elnaghy and Elsaka 2014; Arias et al. 2014; Gagliardi et al. 2015). The recently developed XP4 system (Perfect Medical Instruments Co. Ltd., Shenzhen, China) represents an advanced NiTi rotary instrumentation system featuring a sequential protocol with unique w‐shaped and spoon‐shaped designs, specifically engineered for optimal performance in complex root canal anatomies, including C‐shaped and oval‐shaped configurations. This system features a sequential instrumentation protocol consisting of: (1) an orifice opener (20/.07), (2) a glide path file (15/.04), (3) a longitudinal w‐shaped shaping file (30/.04), and (4) a longitudinal spoon‐shaped finishing file (25/.02). The XP4 instruments incorporate a proprietary thermal treatment process that enhances the NiTi alloy's flexibility and fatigue resistance. Key design innovations include a non‐cutting tip to prevent ledge formation, a conservative taper range for anatomical preservation, and adaptive kinematics that promote efficient debris removal. Despite these technological advancements, there remains a paucity of robust comparative studies evaluating the shaping ability of these NiTi rotary systems when applied to the unique anatomical challenges presented by C‐shaped canal configurations.

Recent advances in 3D printing technique have significantly enhanced endodontic research methodologies through the development of anatomically accurate, standardized tooth replicas (Cui et al. 2018; Zhu et al. 2023; Reymus et al. 2019; Xu et al. 2021; Sekiya et al. 2018). These radiopaque, 3D‐printed models address critical limitations inherent to extracted human teeth specimens, particularly regarding biosafety concerns, ethical constraints, and morphological variability (Zhu et al. 2023). Unlike transparent resin blocks limited to photographic analysis, these advanced replicas allow for nondestructive micro‐computed tomographic (micro‐CT) assessment at micron‐scale resolution, while maintaining specimen integrity for repeated measurements and effectively eliminating interspecimen variability (Zhu et al. 2023). The current study utilized micro‐CT to perform a comparative evaluation of the shaping capabilities of two NiTi rotary systems, XP4 and PTN, in 3D‐printed mandibular molars featuring C‐shaped root canal configurations. The null hypothesis was that there is no significant difference in the shaping ability between the two NiTi rotary systems when used in 3D‐printed mandibular molars featuring C‐shaped root canal configurations.

## 2 Methodology

### 2.1 Specimen Selection

The study protocol has been approved by the Ethics Committee of Ninth People's Hospital of Suzhou with the approval number # KY2022‐089‐01 (Clinical trial number: not applicable). Forty mandibular second molars with fused roots were collected from ethnically Chinese patients requiring tooth extraction due to periodontal disease, non‐restorable carious lesions, or prosthodontic reasons. These teeth retained complete coronal and root anatomy. Each specimen underwent high‐resolution micro‐CT imaging using a SkyScan 1174 system (Bruker‐microCT, Kontich, Belgium). The scanning parameters were set to 50 kV, 800 μA, and a 180° rotation with an incremental step size of 0.7°, achieving an isotropic voxel size of 21.6 μm. Raw scan data were subsequently reconstructed using Mimics 21 (Materialise, Leuven, Belgium).

### 2.2 3D Printing of the C‐Shaped Mandibular Second Molars

Two representative specimens were selected for 3D prototyping based on their distinct canal configurations. Specimen 1 displayed a continuous C‐shaped canal morphology (Melton's Category I), characterized by a patent isthmus extending throughout the entire root length. Specimen 2 exhibited a semicolon‐shaped canal configuration (Melton's Category II) in the coronal and middle thirds. Apically this configuration transitioned into two or three distinct, separated canals (Melton's Category III; Melton et al. 1991), comprising mesiolingual (ML), mesiobuccal (MB), and distal canals.

The tooth replicas were fabricated using a 3D printing protocol as previously described (Zhu et al. 2023). To ensure experimental consistency and minimize variability related to coronal access preparation, digital sectioning was performed 2 mm coronal to the cementoenamel junction (CEJ) using Mimics software. This procedure fully exposed the pulp chamber floor and root canal orifices. The 3D digital models were then converted to STL files and subsequently manufactured using a high‐precision resin‐based 3D printer (Saturn 2, ELEGOO, China), achieving spatial resolutions of 28.5 μm in the *XY* plane and 20 μm along the *Z*‐axis. MOLEGRID Ultradetail photopolymer resin (KEXCELLED, China) was utilized as the printing material. This fabrication process resulted in a total of 32 tooth replicas, with each prototype specimen yielding 16 identical replicas.

### 2.3 Evaluation Fabrication Accuracy of Replica Teeth

To comprehensively assess the accuracy of the 3D‐printed replicas, both trueness (systematic deviation from the reference) and precision (consistency among replicates) were evaluated.

For trueness assessment, the root canal system of each tooth/replica was reconstructed and saved as an STL file. A best‐fit alignment was performed between each replica STL and its corresponding original tooth STL using Geomagic Wrap 2017 (3D Systems, Rock Hill, SC, USA). The aligned surface pairs were imported into CloudCompare software (version 2.11.3) for quantitative deviation analysis. The Dice similarity coefficient (DSC) was computed from the binarized segmentations of the root canal volume of each replica relative to its original tooth, where DSC = 2 |A ∩ B|/(|A| + |B|). This coefficient provides a global measure of spatial overlap. Signed surface distances were then computed from the replica surface to the reference mesh, with positive values indicating outward deviation (overbuild) and negative values indicating inward deviation (underbuild). For each individual replica, the following summary metrics were extracted from its own set of signed distances: mean signed distance (MSD) to indicate overall systematic bias; intra‑replica standard deviation (SD), calculated from signed distances within each replica, to reflect surface variability; average surface distance (ASD) to represent overall bias magnitude; root mean square (RMS) to quantify overall error magnitude; and 95% Hausdorff distance (HD95) to capture the maximum deviation for 95% of the surface while excluding extreme outliers. Signed, color‐coded deviation maps (Figure S1) were also generated for visual inspection of spatial error distribution.

For precision (repeatability) assessment, the inter‑replica variability was quantified as the SD of each of the five metrics across the 16 replicas per specimen group. These inter‑replica SDs reflect the consistency of the printing workflow. Spatial reproducibility was further corroborated by comparing the consistency of color‑coded deviation maps among replicates.

To characterize root canal wall thickness distribution, the external root contour and the root canal contour were segmented from each STL. The minimum distance between these two surfaces was then calculated using CloudCompare, generating color‑coded thickness maps (Figure S2). These maps served two purposes: (1) to verify that the wall thickness distribution of the printed replicas was consistent with that of the original teeth, and (2) to identify the thinnest regions (danger zones) consistently across all replicas, providing a baseline for interpreting subsequent perforation risk and wall‑thinning patterns. Additionally, to compare the cross‑sectional morphology of the root canals, the registered 3D reconstructions of the segmented root canal systems from the prototype teeth and their corresponding replicas were sectioned at three root levels (coronal third, mid‑root, and apical third) using Mimics software. The cross‑sectional shapes were compared between each replica and its prototype, as well as among the 16 replicas to assess inter‑replica consistency (Figure S3).

### 2.4 Root Canal Instrumentation

The 16 resin replicas of each specimen were randomly allocated into two groups (*n* = 8 per group) for root canal preparation using two different NiTi systems according to manufacturers' protocols. All instrumentation procedures were performed by a single operator (Z.X.), a general dentist with 8 years of clinical experience, to eliminate inter‐operator variability. The operator was informed that all replicas featured a C‐shaped configuration. However, the operator was not shown the detailed preoperative micro‐CT images of the two prototype teeth. Prior to the study, the operator underwent specialized hands‐on training for each NiTi system, utilizing additional identical 3D‐printed replicas of the study prototypes, under the direct supervision of an experienced endodontist (Y.G.) with over 27 years of clinical practice. Training continued until consistent and reproducible proficiency was demonstrated, ensuring full adherence to the manufacturers' guidelines.

**Figure 1:**
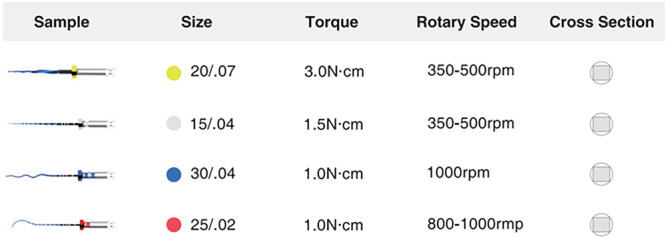
Instrumentation sequence of the XP4 system (as provided by the manufacturer's manual).

All NiTi instrumentation was performed using an X‐Smart Plus endodontic motor (Dentsply Maillefer, Switzerland) with strict adherence to single‐use protocols for all files. The debris on the files was cleaned with wet gauze, and the canal was irrigated with 2 mL of distilled water each time. A decontaminated 30/.04 gutta‐percha point was placed at the WL to confirm that preparation was complete.

a. *Group PTN*: Initial confirmation of apical patency was achieved by passively inserting a #8 and a #10 K‐file (Dentsply Maillefer, Switzerland). Subsequent glide path preparation involved the use of a #15 K‐file up to the predetermined working length, set 1 mm short of the apical foramen. The canal shaping protocol employed a sequence of PTN instruments: X1 (17/.04), X2 (25/.06), and X3 (30/.07). Each instrument was passively introduced into the root canal at a 300 rpm rotation rate and 2.5 N/cm torque. The instruments were used in a slow in‐and‐out motion of approximately 3 mm in amplitude in the apical direction, using a gentle brushing motion against the canal wall. The procedure was repeated twice until the WL was reached.
b. *Group XP4* (Figure 1): The instruments are indicated for continuous rotary motion. The protocol commenced with coronal pre‐flaring using the XP4 Opener (20/.07) at 300 rpm and 3.0 N/cm torque. After verifying apical patency with #8 and #10 K‐files (Dentsply Maillefer, Switzerland), a glide path was established with the XP4 GlidePath file (15/.04) at 300 rpm and 1.5 N/cm torque. Canal shaping was then performed using the XP4 Shaper (30/.04) at 1000 rpm and 1.0 N/cm torque. The shaping process employed gentle pecking motions with axial advancement of 3–5 mm per stroke to progressively reach the working length, while simultaneously creating a taper ranging from 0.04 to 0.08 through repeated movements and extended dwell times. The procedure was finalized with thorough debridement using the XP4 Finisher (25/.02) at 1000 rpm and 1.0 N/cm torque, applying a consistent brushing motion against the recesses of the C‐shaped canal to ensure comprehensive cleaning and shaping. All instrumentation procedures were carried out at a controlled ambient temperature of 22 ± 1°C.

### 2.5 Micro‐CT Analysis of Tooth Replicas

**Figure 2:**
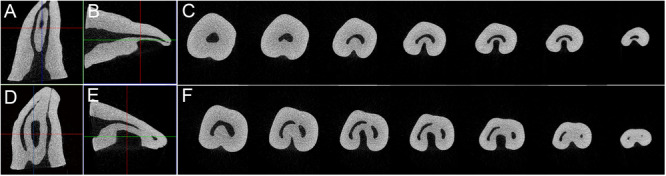
Representative two‐dimensional micro‐CT images of C‐shaped canal replicas demonstrating reduced canal wall thickness beneath radicular grooves. (A–C) A replica with continuous C‐shaped canal configuration (Melton's Category I) along the entire root length: (A) coronal section, (B) sagittal section, and (C) serial axial sections at various root levels. (D–F) A replica exhibiting discontinuous C‐shaped canal configuration (Melton's Category II and III): (D) coronal section, (E) sagittal section, and (F) serial axial sections at different root levels.

**Figure 3:**
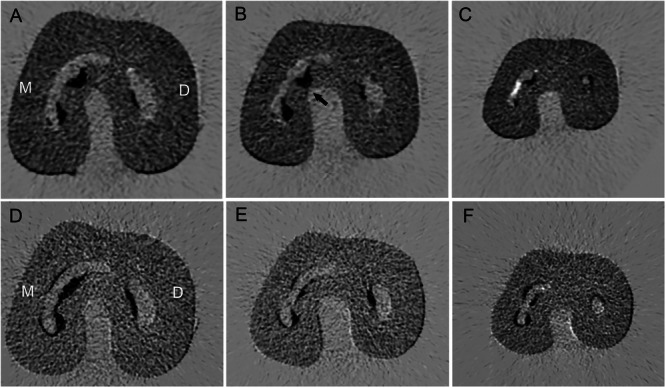
Representative axial micro‐CT cross‐sections of Specimen 2 replicas (Melton's Category II and III) prepared using PTN (top) or XP4 (bottom) systems. (A–C) Superimposed pre‐ and post‐instrumentation images of root canals prepared with the PTN system at the coronal (A), middle (B), and apical (C) root levels. The arrow indicates the site of critical wall thinning (near‐strip perforation). (D–F) Corresponding superimposed pre‐ and post‐instrumentation images prepared with the XP4 system at the coronal (D), middle (E), and apical (F) root levels. D, distal side; M, mesial side. Areas of resin removal are depicted in black.

The resin replicas were scanned using micro‐CT both before (Figure 2) and after root canal instrumentation, following identical scanning protocols. Pre‐ and post‐instrumentation images were geometrically co‐registered using the DataViewer software v1.5.2 (Bruker‐microCT, Kontich, Belgium) (Figure 3), and then the 3D models of the teeth and root canal systems were reconstructed using Mimics software.

To avoid measurement bias, the following geometric parameters associated with root canal shaping were analyzed by another examiner (W.Z.), who was also blinded to the group assignment:

*Canal volume and surface area*: The volumetric and surface area measurements of the root canal system were quantified both before and after instrumentation. The percentage changes in volume and surface area (% ∆) were calculated using the formula: % ∆ = ([*A* − *B*]/*B*) × 100, where *A* represents the post‐instrumentation data and *B* denotes the pre‐instrumentation data.

*Unprepared surface*: The evaluation of the unprepared surface within the root canal was conducted using a Boolean operation in the Mimics software, adhering to the methodology outlined in previous studies (Zhao et al. 2019; Solomonov et al. 2012). Surface voxels that remained unchanged in position from preoperative to postoperative assessments represented the uninstrumented regions of the canal wall. This parameter was quantified as the percentage of static surface voxels relative to the total number of pre‐instrumentation surface voxels, providing a precise measure of the canal areas that remained untouched by the instrumentation process.

*Intra‐observer reliability*: To assess the reproducibility of the unprepared surface measurement, intra‐observer agreement was evaluated using micro‐CT images of 12 randomly selected tooth replicas (three from each experimental group). The unprepared canal surface area (%) was measured twice by the same examiner with a 2‐week interval, following the complete workflow. The intraclass correlation coefficient (ICC) was calculated using a one‐way random effects model for single measures (consistency). The ICC (1, 1) was 0.866 (95% CI: 0.62–0.97, *p* < 0.001), indicating good to excellent intra‐observer reliability. Therefore, the measurement error was considered negligible, and a single measurement was deemed sufficient for subsequent analyses.

*Iatrogenic errors*: Iatrogenic errors including instrument separation and critical wall thinning (near‐strip perforation) were recorded. For the purpose of this study, critical wall thinning approaching strip perforation was operationally defined as a remaining wall thickness of less than 0.1 mm on coregistered axial micro‐CT images, using a conservative threshold similar to those reported in previous studies (Abou‐Rass et al. 1982; Chen et al. 2020). All identified cases were further confirmed on multiplanar and three‐dimensional micro‐CT reconstructions (Figures 3B and Figure S4).

### 2.6 Statistical Analysis

All quantitative data are reported as mean ± SD across the 16 replicas for each specimen group. The HD95 was computed as the 95th percentile of the absolute distance distribution, following the standard definition of the 95% Hausdorff distance. Sample size was determined a priori using G*Power software (version 3.1.9.7; Heinrich‐Heine‐Universität Düsseldorf, Germany). The primary outcomes—percentage changes in canal volume and surface area assessed via micro‐CT—were compared between the two instrumentation protocols (PTN vs. XP4) using independent samples *t*‐tests. The calculation was based on data from a preliminary pilot study conducted under identical experimental conditions. The pilot results indicated a large effect size (Cohen's *d* ≈ 0.85–1.0) accompanied by very low inter‐specimen variability, attributable to the high standardization of the 3D‐printed replicas. Accordingly, a sample size of eight replicas per group (*n* = 8 per group; total *n* = 16 per prototype specimen) was adopted, yielding a total of 32 replicas across the two prototype specimens. This sample size provides sufficient statistical power (> 80%) to detect the primary differences at *α* = 0.05 (two‐tailed). Prior to parametric analysis, the normality of the data distribution was assessed using the Shapiro–Wilk test. All variables, including the percentage changes in canal volume and surface area as well as the percentage of unprepared surface areas, were found to follow a normal distribution (*p* > 0.05). Consequently, all statistical comparisons were performed using Student's independent samples *t*‐test, with the significance level set at *p* < 0.05.

## 3 Results

### 3.1 Trueness and Precision of the Tooth Replicas Prior to Instrumentation

Before instrumentation, the fabrication accuracy of the replicas was evaluated in terms of trueness and precision for the root canal geometry. As shown in Table 1, after best‐fit alignment, the MSD was −0.061 ± 0.011 mm for Specimen 1 and −0.018 ± 0.012 mm for Specimen 2. The negative MSD values indicate a slight inward deviation (i.e., reduced canal dimensions) of the printed replicas (Figure S3). This likely resulted from residual uncured resin not completely washed out from the narrow canal spaces after printing. Such an inward offset is acceptable, as it does not create obstructive protrusions into the canal lumen. The intra‐replica SD values were consistently low (0.031 ± 0.003 mm for Specimen 1 and 0.073 ± 0.015 mm for Specimen 2), demonstrating high precision. The Dice coefficients (0.917 ± 0.012 vs. 0.878 ± 0.032) confirmed high volumetric overlap, while the HD95 and RMS values further supported acceptable overall fabrication fidelity. Representative color‐coded deviation maps (Figure S1) visually confirmed close agreement of canal space between the replicas and the original teeth, with minimal systematic deviation. Canal wall thickness maps (Figure S2) revealed comparable distribution patterns, with the thinnest areas (danger zones) consistently located at the bottom of the radicular grooves in both groups. Baseline measurements of initial canal volume and surface area showed no statistically significant differences across the experimental groups (all *p* > 0.05), with SD substantially lower than those typically reported for extracted teeth, confirming good homogeneity prior to instrumentation.

**Table 1:** Quantitative similarity metrics between original segmented root canal models and 3D‐printed replicas with best‐fit alignment.

	*n*	Dice similarity coefficient	MSD (mm)	Intra‐replica SD (mm)	HD95 (mm)	ASD (mm)	RMS (mm)
Specimen 1 replicas	16	0.917 ± 0.012	−0.061 ± 0.011	0.031 ± 0.003	0.129 ± 0.011	0.069 ± 0.009	0.068 ± 0.010
Specimen 2 replicas	16	0.878 ± 0.032	−0.018 ± 0.012	0.073 ± 0.015	0.221 ± 0.030	0.073 ± 0.011	0.077 ± 0.013

Abbreviations: ASD, average surface distance; HD95, 95% Hausdorff distance (95th percentile); Intra‐replica SD, standard deviation of signed distances across all mesh vertices within each replica (reflecting intra‑replica surface variability); MSD, mean signed distance; RMS, root mean square deviation.

### 3.2 Volume of Resin Removed

In Specimen 1 replicas, the PTN system exhibited significantly less resin removal (2.7 mm^3^, 7.4% of initial volume) compared to XP4 (4.3 mm^3^, 11.6%; *p* < 0.01). This relationship reversed in Specimen 2 replicas, with PTN demonstrating greater volume removal (13.0 mm^3^, 23.1%) than XP4 (8.9 mm^3^, 15.8%; *p* < 0.01) (Table 2).

**Table 2:** Volume, surface area, and unprepared canal surface of C‐shaped canal replicas before and after instrumentation (*n* = 8 per group).

NiTi system	Initial volume (mm^3^)	Final volume (mm^3^)	Volume increase (%)	Initial surface area (mm^2^)	Final surface area (mm^2^)	Surface area increase (%)	Unprepared surface area (%)
Specimen 1 replicas							
PTN	37.1 ± 1.1	39.8 ± 1.7	7.4 ± 2.3	110.8 ± 1.8	111.7 ± 0.9	0.8 ± 1.2	42.0 ± 6.5
XP4	36.6 ± 1.1	40.9 ± 1.3	11.6 ± 3.2**	110.5 ± 1.4	111.8 ± 2.3	1.2 ± 1.2	25.5 ± 8.2**
*t*	0.791	1.370	2.993	0.710	0.127	0.594	4.471
*p*	0.442	0.192	0.001	0.380	0.901	0.462	0.001
Specimen 2 replicas							
PTN	56.2 ± 2.3	69.2 ± 3.9	23.1 ± 7.0	164.1 ± 9.5	176.4 ± 9.1	7.6 ± 4.7	32.0 ± 10.8
XP4	56.8 ± 3.1	65.7 ± 4.1	15.8 ± 5.9*	161.8 ± 5.5	164.5 ± 5.3**	1.7 ± 2.1**	31.6 ± 8.4
*t*	0.391	1.725	2.255	0.599	3.210	3.288	0.088
*p*	0.702	0.107	0.041	0.559	0.006	0.005	0.931

Abbreviation: PTN, ProTaper Next.

* *p* < 0.05

** *p* < 0.01.

### 3.3 Unprepared Canal Surface

**Figure 4:**
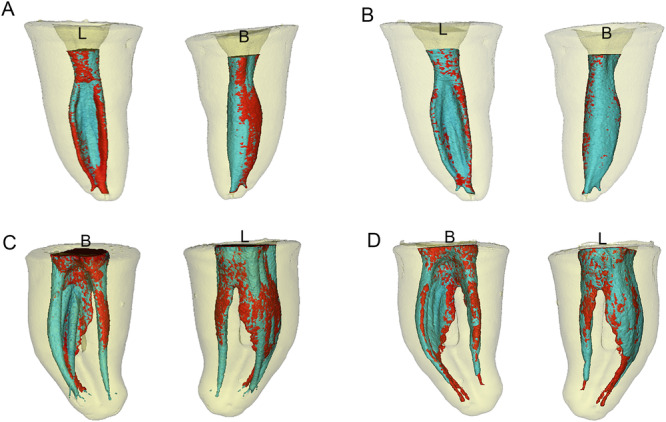
Representative three‐dimensional micro‐CT images of C‐shaped canal replicas before (red) and after (blue) root canal preparation using the PTN or XP4 system. (A) Continuous C‐shaped canal configuration (Specimen 1 replica) following PTN instrumentation; (B) continuous C‐shaped canal configuration (Specimen 1 replica) following XP4 instrumentation; (C) discontinuous C‐shaped canal configuration (Specimen 2 replica) following PTN instrumentation; (D) discontinuous C‐shaped canal configuration (Specimen 2 replica) following XP4 instrumentation. B, buccal view; L, lingual view.

Figure 4 displays 3D reconstructions and segmentations of C‐shaped canal replicas prepared using PTN and XP4 systems. In Specimen 1 replicas, the mean percentage of unprepared canal surface area relative to the total initial surface area was significantly greater in the PTN group (42.0%) than in the XP4 group (25.5%) (*p* < 0.01). Conversely, for Specimen 2 replicas, no statistically significant difference was observed between the two NiTi systems, with mean values of 32.0% for PTN and 31.6% for XP4 (*p* > 0.05) (Table 2).

### 3.4 Iatrogenic Errors

The analysis of instrumentation errors revealed distinct intergroups differences. The PTN group showed no incidence of instrument separation; however, two Specimen 2 replicas developed critical wall thinning (near‐strip perforation) involving both MB and ML canals. In contrast, the XP4 group experienced two instrument separations during root canal preparation, both occurring in Specimen 2 replicas during use of the XP4 Shaper. Notably, no critical wall thinning was identified in the XP4 group. As illustrated in Figure 3 (axial micro‐CT cross‐sections of Specimen 2 replicas), cutting predominantly occurred on the convex canal surface adjacent to the radicular groove. PTN instrumentation tended to cause localized over‐preparation along the MB and ML canal walls, whereas XP4 instrumentation resulted in more uniform cutting patterns with a reduced likelihood of strip perforation.

## 4 Discussion

The intricate anatomical complexity of C‐shaped canals presents substantial clinical and experimental challenges (Jin et al. 2024; Cheung and Cheung 2008; Baghbani et al. 2021; Elnaghy and Elsaka 2014; Elnaghy 2014). The substantial morphological variability in natural teeth often hinders balanced sample allocation in experimental designs. To address these methodological constraints, we utilized 3D‐printed tooth replicas representing two distinct C‐shaped root canal configurations: (1) a continuous C‐shape (Melton's Category I) extending along the entire root length, and (2) a discontinuous C‐shape (Melton's Category II and III) present at varying root levels (Melton et al. 1991). The latter configuration likely results from age‐related changes, where dentine deposition on the walls of C‐shaped canals leads to the formation of separate canals (Gu et al. 2013). Pre‐instrumentation micro‐CT scans and best‐fit deviation analysis demonstrated that the 3D‑printing workflow yielded high anatomical fidelity and minimal inter‑specimen variability (Table 1). The consistently small deviations across all replicas confirmed that the printed models accurately reproduced the target root canal configurations, enabling balanced and reproducible grouping between the two experimental conditions. Consequently, our experimental design not only enhanced the reliability of comparative instrument assessments but also improved sample size efficiency.

Several studies (Vaudt et al. 2009; Guimarães et al. 2017) have consistently shown that uninstrumented canal surfaces persist irrespective of the root canal instrumentation system employed. In the case of C‐shaped canals, despite significant advancements in NiTi instrumentation techniques—including rotary, reciprocating, and eccentric motion systems—a considerable portion of canal surfaces remain unprepared (Solomonov et al. 2012; Yin et al. 2010; Amoroso‐Silva et al. 2017). Clinically, these unprepared regions are problematic as they may impair irrigant penetration and serve as microbial niches, potentially contributing to persistent endodontic infections (Pérez et al. 2020). Recent evidence indicates that adaptive NiTi systems, such as XP‐endo Shaper (FKG Dentaire, Switzerland), offer superior shaping performance with significantly reduced unprepared surface in C‐shaped canal preparation compared to conventional instrumentation techniques (Zhao et al. 2019; Pérez et al. 2020). Furthermore, other innovative designs, such as TRUShape files from Dentsply Sirona, feature a unique longitudinal S‐curve along the file axis, enabling a natural three‐dimensional conforming motion during rotation. This geometry allows the instrument to adapt effectively to non‐round canals—such as oval, flat, or irregular shapes—providing greater wall contact while preserving more dentin and minimizing procedural risks like transportation or perforation (Guimarães et al. 2017). In the present study, we observed distinct differences in instrumentation efficacy between the two tested NiTi systems: PTN showed significantly more unprepared surfaces than XP4 in Specimen 1 replicas (42.0% vs. 25.5%, *p* < 0.01), while both systems performed similarly in Specimen 2 replicas (32.0% vs. 31.6%, *p* > 0.05). Notably, the values observed in our study substantially surpassed those reported by Gazzaneo et al (2021), who documented significantly smaller unprepared areas for both BioRaCe (11.29%) and XP‐Endo Shaper (11.70%) in Type I C‐shaped canals (continuous C). Our data align more closely with Amoroso‐Silva et al (2017), who recorded uninstrumented surfaces of 33% and 30.5% for XP‐Endo Shaper and Reciproc Blue (VDW, Munich, Germany), respectively. However, these results remain markedly lower than the 59.6% (ProTaper) reported by Yin et al. (2010) and 66% (Self‐Adjusting File [SAF; ReDent Nova, Ra'anana, Israel]) by Solomonov et al. (2012). The observed variations may stem from multiple factors, including differences in C‐shaped canal types, instrumentation protocols, operator expertise, baseline anatomical parameters (e.g., volume, surface area, curvature), and the use of tooth replicas versus natural teeth in the experimental design.

The selection of NiTi instrumentation systems should be guided by root canal anatomy to optimize preparation outcomes (Zhu et al. 2023). As shown in Figure 1, the XP4 Shaper features a unique longitudinally w‐shaped (serpentine) configuration. Despite its minimal initial taper of 0.01, the instrument's prolonged intracanal rotational movement enables progressive expansion to the manufacturer‐specified effective taper of 0.04–0.08. While the XP4 Finisher has a longitudinally spoon‐shaped design, acting as an adjunctive instrument specifically designed to improve disinfection and debridement after initial canal preparation. Both instruments share geometric design similarities with the XP‐Endo system (Zhao et al. 2019; Pérez et al. 2020), incorporating modifications to improve wall contact and irrigant agitation, thereby potentially optimizing cleaning efficacy in anatomically complex root canals. However, these NiTi instruments exhibited fundamentally distinct metallurgical behaviors. Unlike the thermo‐responsive XP‐Endo Shaper/Finisher (manufactured using MaxWire technology), which remains straight at room temperature (20°C) but transforms into an S‐shaped configuration at body temperature (37°C), the XP4 Shaper/Finisher maintains a consistent w‐shaped (or spoon‐like) geometry at both room and body temperature. This key difference may significantly impact their clinical performance in C‐shaped canals. As illustrated in Figure 4 and Table 2, the XP4 system exhibited superior operational adaptability in Specimen 1 replicas (characterized by continuous C‐shaped canal configuration with wider isthmus dimensions), providing a significantly greater working space and range of motion compared to the PTN system. This enhanced maneuverability facilitated more effective debridement in anatomically challenging regions (including recess areas and isthmus zones) while maintaining procedural safety, as evidenced by more uniform canal enlargement without increased risk of strip perforation. The wider dimensions of canal space also allowed PTN to follow a straighter apical pathway through isthmus, reducing canal transportation and procedural errors. PTN instruments utilize M‐Wire technology for enhanced flexibility and fatigue resistance, and their off‐centered rectangular cross‐sectional design generates an asymmetric undulating motion, which enhances shaping efficiency and promotes debris displacement (Elnaghy and Elsaka 2014). However, comparative evaluation revealed that PTN exhibits a 1.65‐fold greater unprepared surface area compared to the XP4 system in continuous C‐shaped canal replicas (42.0% vs. 25.5%).

In Specimen 2 replicas with discontinuous C‐shaped canal configurations, where three distinct canals (MB, ML, and distal) were interconnected by narrow isthmus, both NiTi systems achieved comparable proportion of unprepared area. However, notable iatrogenic complications differed between the systems: the PTN group resulted in significantly greater resin removal and two cases of critical wall thinning, while the XP4 Shaper group experienced two instrument separations. The w‐shaped design of the XP4 Shaper posed challenges in navigating the curved MB/ML canals to reach the working length, while the PTN instruments were constrained by their larger diameters (particularly PTN #3) in accessing the narrow isthmus regions. Consequently, no statistically significant difference in the percentage of unprepared surfaces was observed between the systems (*p* > 0.05). Regarding iatrogenic complications, the elevated rotational speed (1000 rpm) and distinctive kinematics of XP4 Shaper resulted in greater frictional resistance and fatigue accumulation within curved, narrow root canal spaces, accounting for two instrument separations (compared to zero with PTN). In contrast, PTN instruments were prone to causing localized over‐preparation along the MB/ML canal walls adjacent to radicular grooves, leading to two cases of critical wall thinning within the danger zones. Key anatomic factors associated with strip perforations include canal curvature severity, dentinal wall thickness, and depth of root concavities or radicular grooves (Tang et al. 2025). These morphological characteristics require particular examination in two specific contexts: their relationship with C‐shaped canal anatomies and their variations across diverse ethnic populations.

Despite the superior shaping performance of the XP4 system in C‐shaped canals, a substantial proportion of the canal surface—25.5% for C1 replicas and 31.6% for C2 replicas—remained uninstrumented. This finding aligns with previous reports that even advanced NiTi systems cannot physically contact every recess, isthmus, or fin within the complex C‐shaped anatomy (Zhao et al. 2019; Solomonov et al. 2012; Yin et al. 2010; Amoroso‐Silva et al. 2017). These unprepared areas represent critical niches where bacterial biofilms and pulpal debris may persist, potentially compromising long‐term treatment success (Pérez et al. 2020). Therefore, mechanical preparation alone is insufficient; chemical irrigation must play an adjunctive yet indispensable role. The use of irrigants such as sodium hypochlorite, delivered with sufficient volume, contact time, and agitation (e.g., sonic or ultrasonic activation), can penetrate into these uninstrumented irregularities and exert antimicrobial and tissue‐dissolving effects (Pérez et al. 2020; Gazzaneo et al. 2021). Notably, the XP4 Finisher, with its spoon‑shaped design, not only debrides but also agitates irrigant more effectively within the C‑shaped space, potentially enhancing chemical disinfection in regions beyond the reach of the shaping file. Nevertheless, even optimal irrigation cannot completely compensate for the lack of mechanical contact in deep isthmus grooves or narrow anastomoses. Thus, future strategies should combine anatomically‑adapted instrument designs with advanced irrigation activation techniques to maximize overall canal cleanliness.

Analysis of volumetric and surface area changes revealed distinct patterns of resin removal among the experiment groups (Table 2). The XP4 system showed significantly greater resin volume removal than PTN in spacious continuous C (Specimen 1) replicas (11.6% vs. 7.4%, respectively; *p* < 0.01). Conversely, PTN demonstrated more resin removal than XP4 in constricted canal space of Specimen 2 replicas (23.1% vs. 15.8%, *p* < 0.01). Surface area measurements revealed that PTN instrumentation of discontinuous C‐shaped canals (Specimen 2 replicas) yielded the most substantial surface area increase (7.6%). Other experimental groups (continuous C/PTN, continuous C/XP4, and discontinues C/XP4) exhibited only marginal changes, ranging from 0.8% to 1.7%. These findings align closely with the 1.7%–6.6% range reported by Amoroso‐Silva et al. (2017). in a comparative study evaluating C‐shaped canal instrumentation using a single‐file reciprocation system (Reciproc; VDW, Munich, Germany) and the SAF system. Notably, these values are significantly lower than the 8.4%–46.5% range documented in numerous previous studies (Gazzaneo et al. 2021; Falcão et al. 2024; Zhao et al. 2025).

Cross‐sectional analysis (Figure 3) revealed preferential resin removal from the convex canal walls adjacent to the lingual radicular groove, resulting in a reduction in canal circumference and enhanced surface smoothness. This effect mitigated the anticipated surface area expansion due to canal enlargement, explaining the minimal change in surface area observed post‐instrumentation. The results underscore the spatial selectivity of dentin/resin removal by different NiTi systems, revealing distinct morphological alterations along concave versus convex canal walls. Collectively, the data establish that both instrument selection and the type of C‐shaped canal are critical determinants of preparation outcomes. This compelling evidence necessitates rejection of the null hypothesis. Our findings provide valuable clinical insights for selecting instruments in C‐shaped canal therapy, emphasizing the need for anatomy‐adapted approaches to minimize procedural risks while optimizing preparation outcomes. The distinct cutting behaviors of two NiTi systems across different categories of C‐shaped canals underscore the critical relationship between instrument design and anatomical preservation in endodontic treatment. Notably, in this study, the comparison conducted herein does not represent a strictly geometry‑matched head‑to‑head evaluation of the two systems under equivalent final apical size and taper conditions. Specifically, the PTN protocol followed the manufacturer's instructions up to instrument X3 (30/.07). In contrast, the XP4 protocol comprised an opener, a glide path file, a 30/.04 shaper, and a 25/.02 finisher, with the shaper capable of generating an effective taper ranging from 0.04 to 0.08 depending on repeated movements and extended dwell time. Thus, this study essentially compared two manufacturer‑recommended clinical protocols rather than the two instrument systems under strictly identical final canal geometries (e.g., same apical diameter and final taper). This disparity may influence the interpretation of volume removal, unprepared surface area, and iatrogenic outcomes. Consequently, readers should avoid overgeneralized mechanistic conclusions attributing the observed differences solely to the intrinsic design features of each instrument, and instead recognize the holistic differences between the two preparation protocols.

This study has several limitations that merit careful consideration. First, the study design was limited to 3D‐printed replicas derived from only two extracted teeth specimens (one continuous and one discontinuous C‑shaped prototype). This may introduce selection bias and constrain the representation of anatomical variability; consequently, the findings should be interpreted as comparisons within these specific configurations rather than as general statements applicable to all continuous or discontinuous C‑shaped canals. Although these models provided typical canal geometries, they may not fully capture the intricate morphological complexities observed in clinical practice, particularly the dynamic changes in canal configuration along the root length that challenge complete debridement and instrumentation (Melton et al. 1991). Second, resin differs from natural dentin in mechanical properties, which may affect instrument performance. Resin is homogeneous and less hard than the heterogeneous, stiffer natural dentin. This likely reduces cutting resistance and yields more uniform friction, potentially lowering torsional and cyclic flexural stresses on flexible NiTi instruments like XP4 during rotation in curved canals. Consequently, the two XP4 separations observed in discontinuous C‑shaped resin replicas may underestimate fracture risk in natural teeth, where higher dentin hardness and irregular tubule structure increase torque accumulation and fatigue. Conversely, the same resin properties may overestimate strip perforation risk, because lower cutting resistance makes it easier for instruments to inadvertently thin or penetrate the canal wall—especially in danger zones near radicular grooves. This could explain why the more flexible XP4 appeared superior in preventing perforation in resin: its adaptability may be less effective against stiffer clinical dentin. Thus, while resin replicas offer standardization and repeatability, the absolute error rates observed should not be directly extrapolated to clinical practice. These methodological limitations require validation via clinical studies or natural tooth models. Nevertheless, this investigation provides valuable preliminary insights and an effective research platform, contributing to our understanding of modern endodontic instrumentation.

## 5 Conclusion

Within the limitations of this ex vivo study using two specific C‐shaped prototypes, the XP4 system showed superior shaping capability with significantly lower unprepared surfaces in spacious continuous C‐shaped canal configurations compared to PTN. In the discontinuous C‐shaped configuration, both systems achieved comparable unprepared surfaces. However, they exhibited distinctly different risk profiles: PTN tended to cause excessive dentin/resin removal leading to critical wall thinning, while XP4 was associated with instrument separations in narrow, curved regions. These results indicate that the relative performance and safety of NiTi systems may vary depending on the specific C‐shaped canal anatomy. Clinicians should carefully consider canal configuration, curvature severity, and dentin thickness when selecting instruments for C‐shaped canal preparation to balance shaping efficiency and procedural safety.

## Author Contributions

Conceptualization: Yongchun Gu and Jia Li. Methodology: Zaidao Xiong, Lu Wang, Wenyuan Zhou, and Zhongyang Shan. Writing and original draft preparation: Zaidao Xiong, Lu Wang, Wenyuan Zhou, Zhongyang Shan, and Chao Liu. Supervision and project administration: Yongchun Gu. All authors have read and agreed to the published version of the manuscript.

## Ethics Statement

The human mandibular second molars used in this study were obtained from the Department of Dentistry, Ninth People's Hospital of Suzhou. All methods have been performed in accordance with the Declaration of Helsinki and have been approved by the Ethics Committee of Ninth People's Hospital of Suzhou with the approval number # KY2022‐089‐01. Informed consent was obtained from all subjects and/or their legal guardian(s).

## Consent

The authors have nothing to report.

## Conflicts of Interest

The authors declare no conflicts of interest.

## Supporting information


**Figure S1:** Color‑coded deviation maps of the original prototype relative to the 3D printed replica after best‑fit alignment, based on signed surface distances. **A** Specimen 1; **B** Specimen 2. Notably, larger deviations (with warm hues) are frequently observed at concave surface areas of the canal, highlighting the sensitivity of such regions to replication error.

**Figure S2:** Representative canal wall thickness maps showing comparable distribution patterns between the original teeth and their replicas. The thinnest regions (danger zones) consistently localized at the bottom of the radicular grooves in both specimens. **A** Specimen 1 prototype; **B** Specimen 2 prototype; **C** Specimen 1 replica; **D** Specimen 2 replica.

**Figure S3:** Superimposed three‑dimensional reconstructions of the segmented root canal systems from the prototype teeth (red) and their corresponding replicas (green), sectioned at different root levels. Panels A–C represent the coronal, middle, and apical cross‑sections of a continuous C‑shaped configuration, respectively, while panels D–F show the corresponding cross‐sections for a discontinuous C‑shaped configuration.

**Figure S4:** Representative micro‐CT images of a replica with discontinuous C‐shaped canal configuration showing critical wall thinning (near‐strip perforation) after root canal preparation with the PTN system. **A** 2D micro‐CT images displaying the sites of critical wall thinning at three orthogonal planes. **B** Three‐dimensional micro‐CT image (lingual view, opaque model). **C** Three‐dimensional micro‐CT image (lingual view; tooth model rendered transparent, with the root canal before preparation shown in blue and after preparation in red). **D** Three‐dimensional micro‐CT image (post instrumentation; horizontal section at the site of critical wall thinning). Arrows indicate the sites of critical wall thinning.

## Data Availability

The original contributions presented in this study are included in the article. Further inquiries can be directed to the corresponding author.
